# Effect and prediction of long-term weather and pollutant exposure on hemorrhagic fever with renal syndrome: based on statistical models

**DOI:** 10.3389/fpubh.2025.1393763

**Published:** 2025-01-31

**Authors:** Weiming Hou

**Affiliations:** Department of Medical Engineering, Air Force Medical Center, PLA, Air Force Medical University, Beijing, China

**Keywords:** hemorrhagic fever with renal syndrome, moving epidemic method, pollutants, time series models, machine learning

## Abstract

**Background:**

Previous studies have typically explored daily lagged relationships between hemorrhagic fever with renal syndrome (HFRS) and meteorology, with a limited seasonal exploration of monthly lagged relationships, interactions, and the role of pollutants in multiple predictions of hemorrhagic fever.

**Methods:**

Our researchers collected data on HFRS cases from 2005 to 2018 and meteorological and contaminative factors from 2015 to 2018 for the northeastern region. First, we applied the moving epidemic method (MEM) to estimate the epidemic threshold and intensity level. Then, we used a distributed lag non-linear model (DLNM) and a generalized additive model (GAM) with a maximum lag of 6 months to evaluate the lagged and interaction effects of meteorological and pollution factors on the HFRS cases. Multiple machine learning models were then applied after Spearman’s rank correlation coefficient analysis was performed to screen for environmental factors in the Northeastern region.

**Results:**

There was a yearly downward trend in the incidence of HFRS in the northeastern region. High prevalence threshold years occurred from 2005 to 2007 and from 2012 to 2014, and the epidemic months were mainly concentrated in November. During the low prevalence threshold period, the main lag factor was low wind direction. In addition, the meteorological lag effect was pronounced during the high prevalence threshold period, where the main lag factors were cold air and hot dew point. Low levels of the AQI and PM_10_ and high levels of PM_2.5_ showed a dangerous lag effect on the onset of HFRS, while extremely high levels of PM_2.5_ appeared to have a protective effect. High levels of the AQI and PM_10_, as well as low levels of PM_2.5_, showed a protective lag effect. The model of PM_2.5_ and the AQI interaction pollution is better. The support vector machine (SVM)-radial algorithm outperformed other algorithms when pollutants are used as predictor variables.

**Conclusion:**

This is the first mathematically based study of the seasonal threshold of HFRS in northeastern China, allowing for accurate estimation of the epidemic level. Our findings suggest that long-term exposure to air pollution is a risk factor for HFRS. Therefore, we should focus on monitoring pollutants in cold conditions and developing HFRS prediction models.

## 1 Introduction

Hemorrhagic fever with renal syndrome (HFRS), also known as epidemic hemorrhagic fever, is a rodent-borne disease caused by various strains of the hantavirus or Seoul virus, characterized by fever, hemorrhage, and acute renal dysfunction (1). As one of the countries most affected by the HFRS epidemic, China has seen a significant decrease in the incidence of HFRS in most regions since 2000.Although preventive measures such as rodent eradication and vaccination have been implemented (2), transient epidemics still occur at certain times and in specific regions.

Early assessments of epidemic thresholds and risk classification focused on influenza and respiratory infections (3, 4), which have proven novel in application and effective for infectious diseases in China. However, there is a lack of relevant studies on HFRS. Earlier studies have suggested that climatic factors may contribute to the incidence of HFRS. According to an epidemiological survey in 2002, rainfall was identified as a predictor of HFRS transmission in the epidemic source (r = −0.63) (5). Furthermore, several studies have gradually refined the understanding of the relationship between meteorological factors and HFRS, highlighting varying effects in terms of lag and dose–response relationships. For example, in Nei Menggu province, Wen-Yi Zhang et al. found that rainfall, land temperature, and humidity were associated with HFRS onset at a lag of 3–5 months, after controlling for autocorrelation, seasonality, and long-term trends (6). Recent studies have also shown that wet and warm climatic conditions in the northeastern favor the occurrence and growth of HFRS (7). However, there is limited variability in climatic factors across different epidemic risk classifications. In addition, HFRS may be associated with air pollutants in terms of incidence because it is partly transmitted via the aerosol route. However, although several studies have confirmed the lag and correlation with air pollution in infectious diseases, few studies have been conducted on HFRS (8, 9).

The overall goal of this study was to explore the epidemiological characteristics of HFRS, the graded warning system, the lag and interaction effects of climate and pollutants, and the subsequent development of models for predicting HFRS outbreaks. Our specific objectives were to (a) calculate the epidemic thresholds and assess the risk levels, (b) explore the effects of lags and interactions of meteorological and pollution factors, and (c) construct stratified models for HFRS onset, selecting appropriate models for different populations.

## 2 Materials and methods

### 2.1 Setting

Supplementary Figure S1 shows the geographical location of the study area—Heilongjiang, Jilin, and Liaoning provinces. The three provinces are located in the northeastern of China and have medium levels of economic development and population size.

### 2.2 Data collection

We obtained HFRS case surveillance data from the National Public Health Data Center of China^1^ for the study area covering the period from 2005 to 2018. All patients were diagnosed according to the HFRS management criteria issued by the Ministry of Health of the People’s Republic of China. We obtained the corresponding daily weather data, including air temperature and dew point temperature, from the China Meteorological Data Sharing Service (data.cma.cn). Pollutant information, including CO, NO_2_, and O_3_, was originally sourced from the National Oceanic and Atmospheric Administration (NOAA).

### 2.3 Estimation of the epidemic threshold and intensity level

We used the R language implementation of the moving epidemic method (MEM) (package “mem”), which is available online for free. The method is based on a complex mathematical algorithm that can be summarized in three steps. The first step is the division of the pre-epidemic, epidemic, and post-epidemic periods. In the second step, the pre- and post-epidemic values of the historical seasons are used to calculate the baseline and epidemic thresholds. In the third step, the maximum values of n surveillance indicators during the epidemic period are selected separately to calculate different epidemic intensity thresholds. The unilateral 50%CI upper limit of the geometric mean of the n maximum surveillance indicators during the epidemic period is defined as the medium intensity threshold, the unilateral 90%CI upper limit as the high-intensity threshold, and the unilateral 95%CI upper limit as the very high-intensity threshold.

### 2.4 The lagging and interaction effect of DLNM and GAM

Distributed lag non-linear models (DLNM) have been widely used to assess the exposure–lag–response relationship between environmental factors and human diseases such as congenital heart disease, hand, foot, and mouth disease, and chronic sinusitis (8, 10–12). The model can be written as follows:


$$\begin{array}{l} log\left[E\left(Y_{t}\right)\right]=\alpha_{1}+NS\left(M,df\mathrm{,lag,}df\right)+\sum NS\left(X_{t}\right)+\sum \left(X_{t}\right)+\\ NS\left(Time,df\right)+\beta Month_{t} \end{array}$$

To analyze the lag and extreme effects of climate factors, air temperature, dew point temperature, wind direction, and wind speed were considered and applied to the cross-basis functions of a DLNM. Here, *Y_t_* is the number of the HFRS cases in monthly *t*; *α_1_* is the intercept of the entire model; *NS* is a natural cubic spline that acts as a smooth function of the model; *M* represents the estimated climate or pollutants variable related to HFRS; and *X_t_* represents other climate and pollutant variables involved in the pathogenesis of HFRS, for which non-linear confounding effects are adjusted. When constructing the meteorological factor model, $\sum \left(X_{t}\right)$ does not exist, whereas in the pollution model, meteorological factors are used as confounding factors to construct $\sum \left(X_{t}\right)$ . The *NS* was applied to adjust for the monthly confounding effects in the model. *Month* is a binary variable used to control the effect of time, and *β* represents regression coefficients. The optimal degrees of freedom (*df*) for the spline function were estimated using the Akaike information criterion for quasi-Poisson (Q-AIC) and minimum partial regression coefficient (PACF_min_) criteria. The *NS* with 4 *df* was used for the climate factors, except for wind direction, which used 5 df during the period of low epidemic intensity. For both the high epidemic intensity period and the overall model, the NS with 4 df was applied to the climate and pollutant factors. The lag space was set to 3 *df*. The *NS* with 2–3 *df/*year was applied to the time variable in both pollutant and climate models. The climate model was constructed using the glm () function, while the pollution model was constructed using the gam () function.

Subsequently, a generalized additive model (GAM) was used to explore the interaction between the pollutants and the prevalence of HFRS. The model formula can be written as follows:


$$\log\left[E\left(Y_{t}\right)\right]=\alpha_{2}+s\left(X_{1},X_{2}\right)+s\left(X_{3}\right)+\sum \left(X_{t}\right)$$

*α_2_* is the intercept; *X_1_* represents the AQI, whereas *X_2_* and *X_3_* denote the other two pollutants. *s ()* indicates a penalized spline function. *s* (*X_1_*, *X_2_*) represents the spline function for the interaction between the parameters *X_1_* and *X_2_*. *X_1_*, *X_2_,* and *X_3_* represent 6-month lagged variables. $\sum \left(X_{t}\right)$ represents the factors of climate.

### 2.5 Construction of a prediction model in GPR and SVM

A Gaussian process (GP) can be regarded as an extended function of a multivariate Gaussian distribution, which can be applied to a wide range of variables. In a Gaussian process (GP), it is assumed that any finite set of data follows a multivariate Gaussian distribution. Prior beliefs concerning the relationships between variables are incorporated into these (an infinite number of) multivariate Gaussian distributions to create a model that represents the observational variance. The combination of multiple Gaussian distributions in a GP can effectively model non-linear relationships and is more versatile than traditional parametric models, which depend on fitting a global model. This is because multivariate Gaussians can represent local covariance patterns between individual sites (13).

Support vector machines (SVMs) are a non-probabilistic binary linear regression method. Given a set of training data labeled as belonging to one of two classes, the algorithm maps the data into a space and defines a hyperplane that maximizes the margin between the two classes to separate them. This plane is called the “maximal marginal hyperplane.” An algorithm uses a kernel approach to acquire non-linear mapping to the feature space if linear integration is impossible. Thus, the hyperplane of the feature space stands for the non-linear boundary of the determination in the input space (14). All model metrics are compared using traditional machine learning metrics such as RMSE, R^2^, and MAE (15–17). A total of 75% of the dataset is used as the training set, while the remaining 25% is used as the test set. All analyses in our study were performed using R software (version 4.1.3).

## 3 Results

### 3.1 HFRS surveillance in northeastern China

A total of 59,431 HFRS cases were reported in the three eastern provinces of China from 2005 to 2018, showing a decreasing trend each year (Table 1). This was followed by the main epidemic area in Heilongjiang province, with a total of 28,074 cases until 2018. The incidence of influenza was primarily observed in the individuals aged 15–39 and 40–59 years, accounting for 86.42% of all cases.

**Table 1:** Distribution of the HFRS cases by age groups, region, and season in northeastern China, 2005–2018.

Characteristic		0–14	15–39	40–59	≧60	Total	Population (10^4^)	Incidence (10^−2^%)
		No. of the HFRS cases (%)						
Year	2005	245(2.26%)	5,148(47.54%)	4,586(42.35%)	850(7.85%)	10,829	10,757	1.01
	2006	138(1.8%)	3,680(47.98%)	3,310(43.16%)	542(7.07%)	7,670	10,917	0.7
	2007	60(1.17%)	2,386(46.64%)	2,268(44.33%)	402(7.86%)	5,116	10,952	0.47
	2008	30(0.85%)	1,519(43.29%)	1,637(46.65%)	323(9.2%)	3,509	10,874	0.32
	2009	31(0.93%)	1,313(39.42%)	1,651(49.56%)	336(10.09%)	3,331	10,907	0.31
	2010	36(1.2%)	1,178(39.21%)	1,432(47.67%)	358(11.92%)	3,004	10,955	0.27
	2011	38(1.17%)	1,162(35.91%)	1,630(50.37%)	406(12.55%)	3,236	10,966	0.3
	2012	54(1.51%)	1,283(35.76%)	1737(48.41%)	514(14.33%)	3,588	10,973	0.33
	2013	52(1.33%)	1,311(33.5%)	1973(50.41%)	578(14.77%)	3,914	10,976	0.36
	2014	45(1.15%)	1,228(31.36%)	1992(50.87%)	651(16.62%)	3,916	10,976	0.36
	2015	28(0.93%)	895(29.7%)	1,538(51.05%)	552(18.32%)	3,013	10,947	0.28
	2016	17(0.66%)	699(27.11%)	1,384(53.69%)	478(18.54%)	2,578	10,910	0.24
	2017	36(1.3%)	743(26.81%)	1,432(51.68%)	560(20.21%)	2,771	10,875	0.25
	2018	32(1.08%)	784(26.52%)	1,478(50%)	662(22.4%)	2,956	10,836	0.27
Region	Heilongjiang	344(1.23%)	11,459(40.82%)	13,018(46.37%)	3,253(11.59%)	28,074	3,819	7.35
	Jilin	176(1.33%)	5,388(40.71%)	6,252(47.24%)	1,418(10.71%)	13,234	2,736	4.84
	Liaoning	322(1.78%)	6,482(35.77%)	8,778(48.44%)	2,541(14.02%)	18,123	4,362	4.15
Season	Spring (March–May)	270(1.69%)	6,660(41.63%)	7,322(45.77%)	1745(10.91%)	15,997		
	Summer (June–August)	126(1.01%)	4,824(38.68%)	6,004(48.14%)	1,518(12.17%)	12,472		
	Autumn (September–November)	230(1.37%)	6,213(36.93%)	8,139(48.38%)	2,241(13.32%)	16,823		
	Winter (December–February)	216(1.53%)	5,632(39.83%)	6,583(46.56%)	1708(12.08%)	14,139		
Total		842(1.42%)	23,329(39.25%)	28,048(47.19%)	7,212(12.14%)	59,431	10,917	5.44

**Figure 1:**
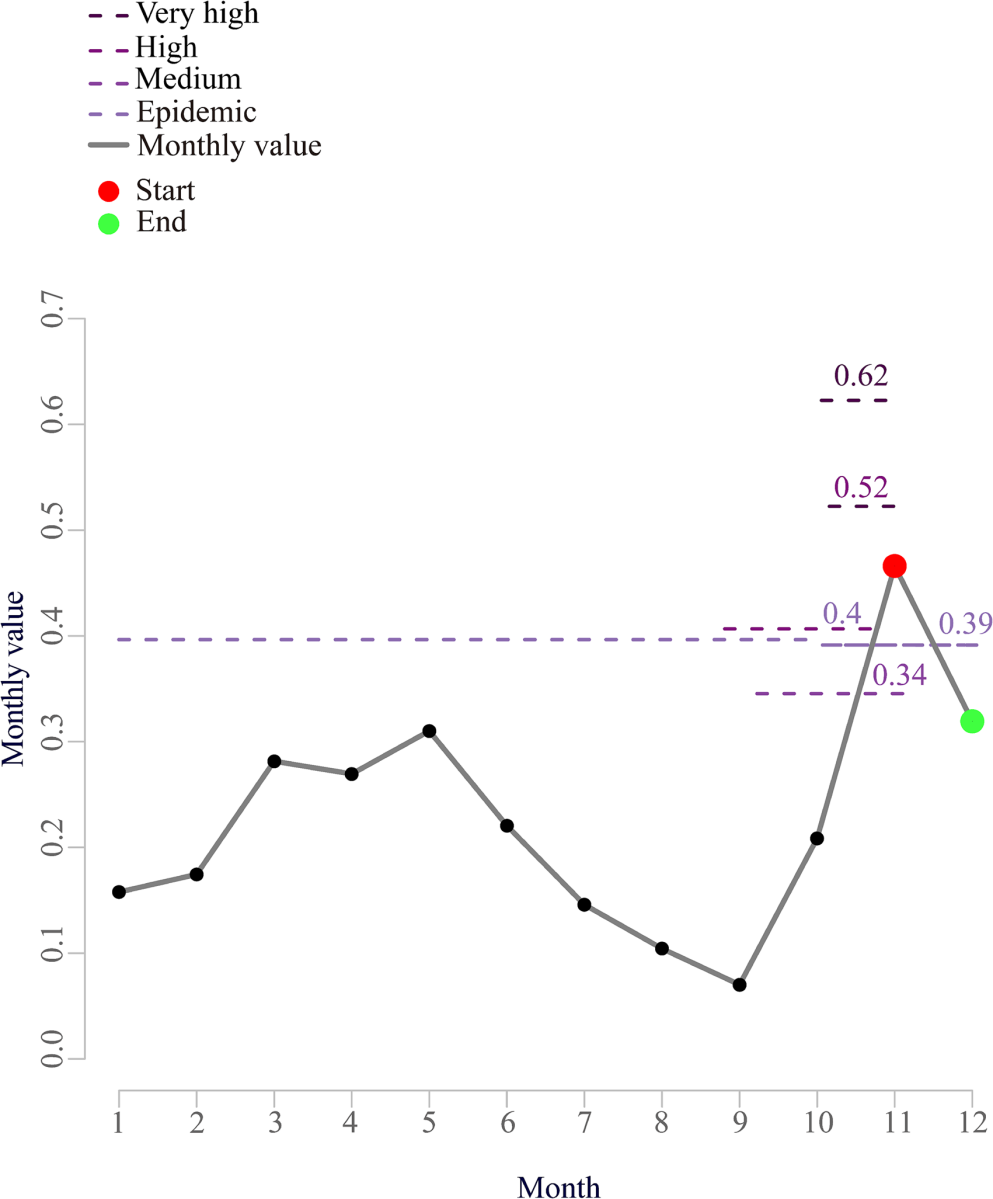
Surveillance and early warning of HFRS in northeastern China during 1–12 months in 2018.

Based on Table 1, however, there was a short-term rise in the cases from 2012 to 2014. We also performed a calculation of the prevalence threshold and determined from Supplementary Table S1 that the optimal parameter *δ* was 7.0 after the calculation of the popular threshold model. As shown in Table 2, the years with a high prevalence threshold were 2005–2007 and 2012–2014, while the years with a low prevalence threshold were 2008–2011 and 2015–2018. Based on the threshold model prediction shown in Table 2 and Figure 1, it was concluded that the epidemic months were primarily concentrated in November.

**Table 2:** Characteristics of the peak values in each year used in the model.

Year	Peak (per 10^−5^)	Peak month	Epidemic threshold	Threshold intensity			Level	Series
				Medium	High	Very high		
2005	1.57	11	0.46	0.46	0.64	0.80	Very high	High
2006	1.03	11	0.46	0.46	0.74	0.98	Very high	High
2007	0.81	11	0.40	0.41	0.77	1.02	High	High
2008	0.47	11	0.46	0.46	0.81	1.08	Medium	Baseline
2009	0.44	6	0.47	0.47	0.81	1.07	Baseline	Baseline
2010	0.57	11	0.47	0.47	0.80	1.07	Medium	Baseline
2011	0.43	11	0.46	0.46	0.65	0.83	Baseline	Baseline
2012	0.65	11	0.46	0.46	0.55	0.68	High	High
2013	0.60	11	0.38	0.38	0.50	0.60	High	High
2014	0.55	11	0.38	0.38	0.51	0.62	High	High
2015	0.42	11	0.39	0.39	0.52	0.64	Medium	Baseline
2016	0.41	11	0.40	0.40	0.52	0.62	Medium	Baseline
2017	0.35	11	0.39	0.39	0.53	0.64	Baseline	Baseline
2018	0.47	11	0.40	0.40	0.52	0.62	Medium	Baseline

### 3.2 Exposure–response relationships and lagging effect for the climate factors

The summary statistics for all HFRS cases and environmental variables in northeastern China are shown in Supplementary Table S2. The Spearman’s rank correlation coefficient analysis showed that HFRS was significantly correlated with air temperature (r = −0.18, *p* < 0.05), dew point temperature (r = −0.23, *p* < 0.01), wind direction (r = 0.22, *p* < 0.01), and wind speed (r = 0.29, *p* < 0.01) (Supplementary Table S3). As shown in Supplementary Figure S2, these climate factors were associated with high relative risk at the lags above moderate levels, except for air temperature.

From the dose–response relationship shown in Supplementary Figure S3, air temperature showed mostly a U-shaped relationship with the risk of HFRS, both in general and across the different regions and age groups, while the other factors mostly showed an arch bridge-shaped relationship. In Liaoning province, air temperature, dew point temperature, and wind speed all showed a parabolic decreasing trend in their relationship with HFRS risk. As shown in Supplementary Table S5, the climate lag effect was weak during the low prevalence threshold period, with sensitivity mainly concentrated in the high prevalence areas of Heilongjiang province and the 0–14 years age group, where the main lag factor was low wind direction. As shown in Supplementary Table S6, the meteorological lag effect was higher during the high prevalence threshold period, with sensitivity mainly concentrated in the 0–14 years and 60 years and above age groups, where the main lag factors were cold air and hot dew point. When comparing the climatic lags during the low and high prevalence threshold periods (Supplementary Tables S5, S6), we found that low wind direction and windy conditions showed a dangerous lag effect on HFRS onset (OR > 0), while high wind direction and windless conditions showed a protective lag effect (OR < 0). In addition, air temperature showed protective effects at both low and high levels, while cold air showed a dangerous effect in the 0–14 years age group during the high prevalence threshold period (OR (95% CI): 3.2e+17(8.4e+08, 1.2e+26)). Cold dew point had a little lag effect, while hot dew point showed a protective effect during the low prevalence threshold period. However, this effect was reversed during the high prevalence period.

### 3.3 Exposure–response relationships and lagging effect for the pollutants

**Figure 2:**
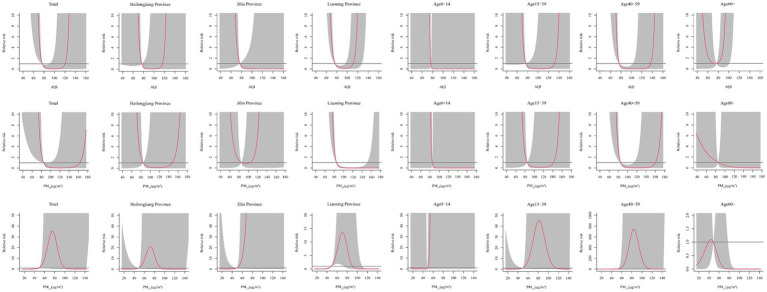
Effect of the different pollutants on the incidence of HFRS across the different months for total, regions, and age groups.

**Figure 3:**
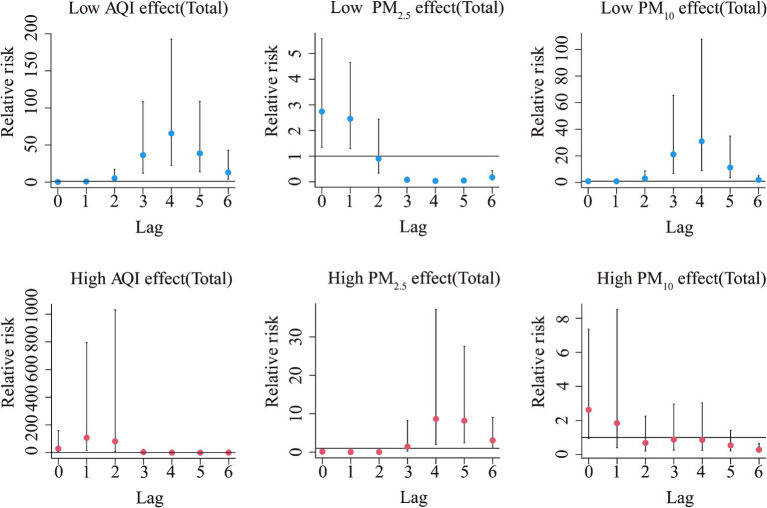
Summary of the estimated extreme effects at the 5th and the 95th percentile of the pollutants on the HFRS cases for the total during the different lag months. The median value of each pollutant (AQI: 76.79, PM_2.5_: 48.7 μg/m^3^, PM_10_: 84.89 μg/m^3^) serves as a reference level.

The Spearman’s rank correlation coefficient analysis showed that HFRS was significantly correlated with the AQI (r = 0.40, *p* < 0.05), PM_2.5_ (r = 0.37, *p* < 0.05), and PM_10_ (r = 0.40, *p* < 0.01) (Supplementary Table S4). As shown in Supplementary Figure S4, these factors were associated with high relative risk at the lags above high levels, except for PM_10_. From the dose–response relationship shown in Figure 2, PM_2.5_ mostly showed an arch bridge-shaped relationship, while the AQI and PM_10_ mostly showed a U-shaped relationship with the risk of HFRS, both in general and across the different regions and age groups. In Jilin province and the 0–14 years age group, the AQI exhibited a parabolic decreasing trend, while PM_2.5_ showed a parabolic increasing trend. As shown in Figure 3, in terms of the total pollution lags, the effects of the low-level pollutants were mainly concentrated in the long-term lag conditions (3–6 months), while the effects of the high-level pollutants were mainly concentrated in the short-term lag conditions (1–2 months). In terms of the lagging trend, PM_2.5_ differed from the other pollution factors. As shown in Table 3, except for high-level PM_10_, the lag effect of the other pollution factors was more pronounced, and the sensitivity was mainly concentrated in Liaoning province and the age group of 40–59 years. Among these, we found that low levels of the AQI and PM_10_ and high levels of PM_2.5_ showed a dangerous lag effect on the onset of HFRS (OR > 0), while extremely high levels of PM_2.5_ (P95) showed a protective effect. In addition, high levels of the AQI and PM_10_ and low levels of PM_2.5_ showed a protective lag effect (OR < 0). However, at extremely high levels of the AQI (P95), a dangerous effect was observed.

**Table 3:** The cumulative effects of the extreme pollutant factors on the HFRS cases by region and age group.

Series	Variables	Cumulative effects (95%CI)					
		Low AQI effect	High AQI effect	Low PM_2.5_ effect	High PM_2.5_ effect	Low PM_10_ effect	High PM_10_ effect
	Total cases	**7.8e+05(78.501, 7.7e+09)** **7.5e+03(15.225, 3.7e+06)**	0.051(0.002, 1.323) 1e+04(0.003, 3.3e+10)	**1.1e−04(7.2e−08, 0.182)** **0.004(0.000, 0.384)**	22.119(0.692, 707.182) 0.067(0.000, 1.8e+04)	**4e+04(7.046, 2.3e+08)** **1.1e+03(3.266, 3.7e+05)**	0.070(0.004, 1.248) 0.364(0.000, 1.3e+04)
Region	Heilongjiang	4.8e+06(0.808, 2.8e+13) 2.4e+04(0.628, 8.9e+08)	0.046(0.000, 13.048) 7.5e+05(0.000, 8.5e+16)	0.000(0.000, 29.414) 0.003(0.000, 10.229)	17.086(0.041, 7110.833) 0.002(0.000, 5.1e+06)	4743.176(0.003, 7.3e+09) 257.344(0.018, 3.8e+06)	0.147(0.001, 16.933) 2.665(0.000, 8.4e+07)
	Jilin	3.2e+04(0.031, 3.3e+10) 1.1e+03(0.101, 1.3e+07)	0.029(0.000, 3.540) 0.005(0.000, 1.5e+07)	0.001(0.000, 44.948) 0.010(0.000, 10.270)	38.629(0.245, 6.1e+03) 9.2e+03(0.000, 6.5e+11)	185.119(0.002, 1.7e+07) 24.452(0.011, 5.3e+04)	0.948(0.020, 44.050) 2.9e+04(0.028, 3e+10)
	Liaoning	**1.6e+05(844.167, 3.1e+07)** **2.4e+03(70.763, 8.3e+04)**	**0.128(0.021, 0.802)** **1.1(221.522, 5.4e+09)**	**1.6e−04(2.5e−06, 0.010)** **0.005(0.000, 0.064)**	**12.214(1.754, 85.054)** **0.001(0.000, 0.988)**	**4.7e+05(693.362, 3.2e+08)** **6478.497(80.564, 5.2e+05)**	**0.018(0.002, 0.160)** 0.001(0.000, 1.786)
Age group	0–14 years	3.9e+17(0.000, 1.4e+57) 0.415(0.000, 7.7e+38)	0.000(0.000, 5.3e+09) 0.000(0.000, 3.8e+81)	0.000(0.000, 3.9e+24) 0.000(0.000, 2.7e+15)	6.2e+05(0.000, 2.3e+23) 1.3e+21(0.000, 1.1e+103)	4.4e+14(0.000, 1e+61) 7.2e+09(0.000, 1.2e+41)	0.000(0.000, 1.4e+11) 0.000(0.000, 5.9e+52)
	15–39 years	4.9e+05(0.775, 3.1e+11) 6.1e+03(0.748, 4.9e+07)	0.034(0.000, 3.569) 51.177(0.000, 7.9e+10)	0.001(0.000, 24.237) 0.009(0.000, 7.870)	18.395(0.130, 2.6e+03) 3.181(0.000, 1.4e+08)	5.1e+04(0.009, 2.9e+11) 1192.604(0.034, 4.2e+07)	0.099(0.001, 17.719) 11.978(0.000, 1.2e+09)
	40–59 years	**1e+07(336.722, 3.1e+11)** **4.7e+04(45.058, 4.9e+07)**	**0.012(0.000, 0.460)** 14.229(0.000, 2e+08)	**6.6e−06(1.7e−09, 0.026)** **0.001(0.000, 0.104)**	**124.231(2.633, 5860.926)** 29.291(0.000, 2.7e+07)	**2.7e+05(107.071, 6.6e+08)** **3812.795(19.754, 7.4e+05)**	**0.045(0.003, 0.614)** 0.448(0.000, 5.8e+03)
	60 years and above	50.471(4e−03, 5.9e+05) 7.647(0.014, 4.3e+03)	11.547(0.368, 362.732) **1.8e+15(1.6e+08, 2e+22)**	0.223(0.000, 427.895) 0.576(0.005, 69.983)	0.105(0.003, 4.041) **5.8e−12(7.6e−18, 4.4e−06)**	5.465(0.000, 1.1e+09) 3.431(0.000, 1.3e+06)	0.376(0.001, 223.061) 0.008(0.000, 1.8e+08)

Bold font indicates statistical significance at the 0.05 level.

### 3.4 Interaction and comparison of the multiple pollutant models

**Figure 4:**
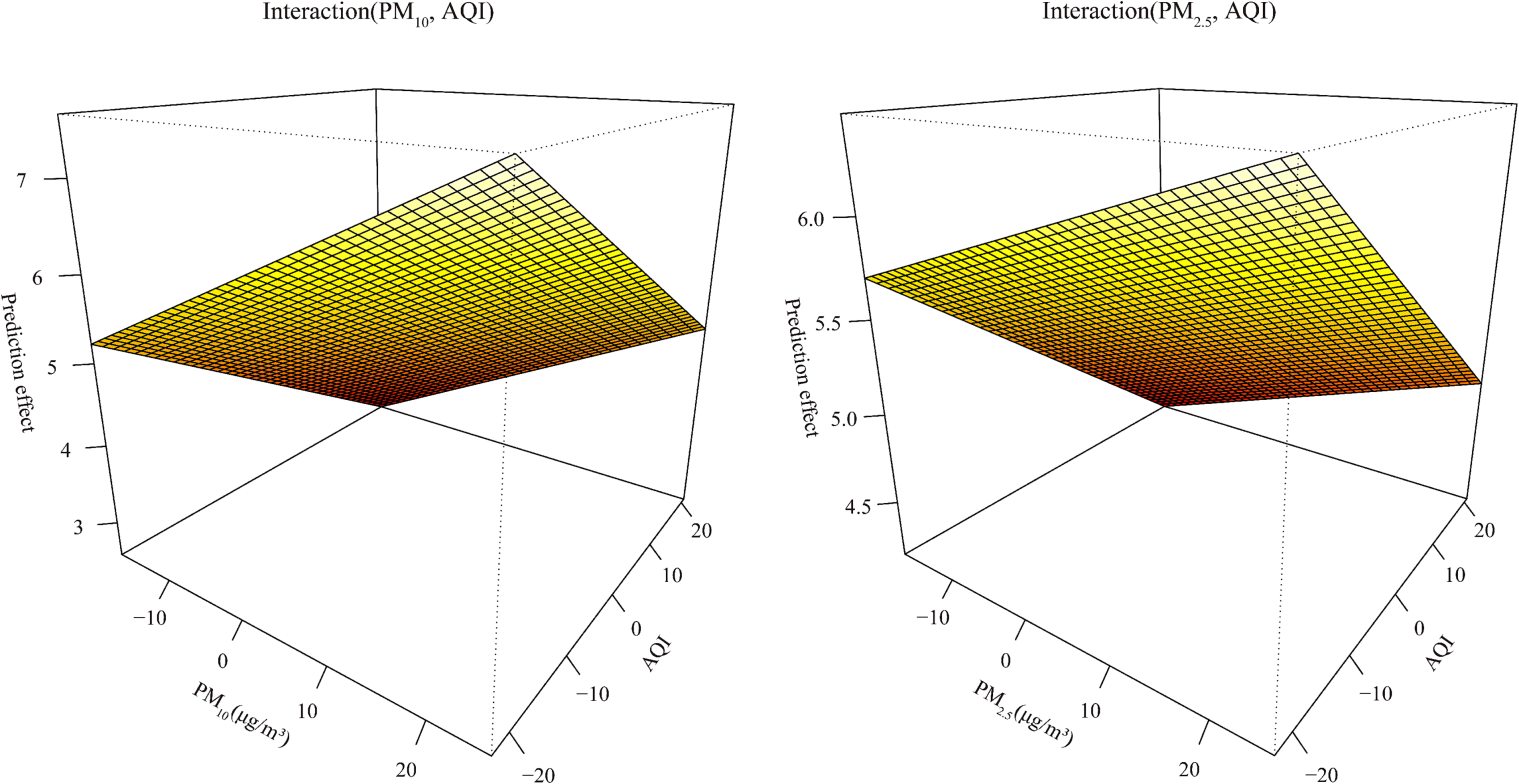
The fitting interactions of the association between the pollutants and HFRS cases in northeastern China during 2015–2018 based on the generalized additive model (GAM).

From Supplementary Figure S5, we can see that the AQI interacted with PM_2.5_ and PM_10_ in relation to HFRS incidence. PM_10_ was weakly positively correlated with the risk of HFRS, while PM_2.5_ showed the opposite relationship. From the interaction effect shown in Figure 4, we found that low AQI combined with high levels of PM_2.5_ and PM_10_ had the greatest impact on HFRS onset. The results from the test in Supplementary Table S7 indicate that the model involving the interaction between PM_2.5_ and the AQI performed better (R^2^ = 44.1%). From Supplementary Table S8 and Table 4, the model fit was best in Liaoning province among the different regions (R^2^ > 70%) and in the 15–39 age group. In addition, the GPR model showed the same fit as that of the SVM model. In the GPR model, the prediction results were good, except for the polydot kernel function. In the SVM model, good prediction results were observed with the radial and sigmoid kernel functions. Based on the SVM-radial model for exploring the importance of the variables related to HFRS, the priority order was the pollutant factors (in the order of the AQI, PM_10_, and PM_2.5_), followed by the climatic factors (in the order of windspeed, dew point temperature, and air temperature).

**Table 4:** Comparison of the prediction results with the different kernels of the support vector machine (SVM) models.

Model	Series		Parameters	cv.fold	Training set			Test set		
					RMSE	R^2^	MAE	RMSE	R^2^	MAE
SVM (Linear)		Total cases	cost = 10,gamma = 0.143	10	60.657	0.687	32.258	84.242	0.086	70.695
	Region	Heilongjiang	cost = 10,gamma = 0.143	10	39.826	0.684	18.846	54.348	0.007	41.752
		Jilin	cost = 5,gamma = 0.143	10	10.986	0.712	6.546	16.699	0.022	12.518
		Liaoning	cost = 10,gamma = 0.143	10	14.752	0.756	9.796	30.271	0.335	24.729
	Age group	0–14 years	cost = 0.1,gamma = 0.143	10	2.097	0.193	1.510	1.892	0.085	1.325
		15–39 years	cost = 10,gamma = 0.143	10	14.014	0.790	8.570	17.932	0.386	14.841
		40–59 years	cost = 10,gamma = 0.143	10	30.523	0.696	17.297	44.173	0.105	35.462
		60 years and above	cost = 0.1,gamma = 0.143	10	22.295	0.197	14.206	17.911	0.092	13.482
SVM (Polynomial)		Total cases	degree = 3,cost = 4,gamma = 0.143	10	69.488	0.585	38.329	76.208	0.114	64.232
	Region	Heilongjiang	degree = 3,cost = 1,gamma = 0.143	10	58.073	0.429	32.649	43.421	0.077	30.560
		Jilin	degree = 3,cost = 4,gamma = 0.143	10	11.554	0.680	6.786	16.653	0.020	12.378
		Liaoning	degree = 3,cost = 4,gamma = 0.143	10	17.466	0.655	11.811	30.125	0.354	23.966
	Age group	0–14 years	degree = 3,cost = 0.1,gamma = 0.143	10	2.097	0.193	1.510	1.892	0.085	1.325
		15–39 years	degree = 3,cost = 3,gamma = 0.143	10	17.890	0.656	11.781	15.327	0.449	13.408
		40–59 years	degree = 3,cost = 2,gamma = 0.143	10	39.535	0.499	24.128	38.274	0.182	29.505
		60 years and above	degree = 3,cost = 0.1,gamma = 0.143	10	22.295	0.197	14.206	17.911	0.092	13.482
SVM (Radial)		Total cases	cost = 1,gamma = 0.5	10	66.263	0.705	39.039	75.872	0.103	59.891
	Region	Heilongjiang	cost = 1,gamma = 0.5	10	49.024	0.695	25.009	48.785	0.007	34.382
		Jilin	cost = 1,gamma = 0.5	10	10.968	0.767	6.570	15.864	0.009	11.854
		Liaoning	cost = 1,gamma = 1	10	11.380	0.897	7.870	31.249	0.404	26.547
	Age	0–14 years	cost = 1,gamma = 4	10	1.135	0.800	0.567	2.017	0.000	1.524
		15–39 years	cost = 1,gamma = 1	10	13.255	0.879	7.932	15.210	0.453	13.080
		40–59 years	cost = 1,gamma = 1	10	26.272	0.856	15.701	44.812	0.043	32.347
		60 years and above	cost = 1,gamma = 2	10	12.728	0.808	5.716	19.829	0.004	15.141
SVM (Sigmoid)		Total cases	coef0 = 0.1,gamma = 0.5	10	66.263	0.705	39.039	75.872	0.103	59.891
	Region	Heilongjiang	coef0 = 0.1,gamma = 0.5	10	49.024	0.695	25.009	48.785	0.007	34.382
		Jilin	coef0 = 0.1,gamma = 0.5	10	10.968	0.767	6.570	15.864	0.009	11.854
		Liaoning	coef0 = 0.1,gamma = 1	10	11.380	0.897	7.870	31.249	0.404	26.547
	Age	0–14 years	coef0 = 0.1,gamma = 4	10	1.135	0.800	0.567	2.017	0.000	1.524
		15–39 years	coef0 = 0.1,gamma = 1	10	13.255	0.879	7.932	15.210	0.453	13.080
		40–59 years	coef0 = 0.1,gamma = 1	10	26.272	0.856	15.701	44.812	0.043	32.347
		60 years and above	coef0 = 0.1,gamma = 2	10	12.728	0.808	5.716	19.829	0.004	15.141

## 4 Discussion

In the European Centre for Disease Prevention and Control (ECDC), the MEM is a standardized approach for epidemiological classification and early warning of infectious diseases (18). However, the application is limited to diseases with a yearly upward trend, such as influenza and hand, foot, and mouth disease. The better-controlled infectious diseases, such as HFRS, have limited application in epidemic grading. Based on recent global environmental pollution and the short-term annual rise in hemorrhagic fever cases, this study applied the MEM to classify and issue warnings regarding its epidemic status. As the MEM was originally applied to weekly cases, monthly data were used in this study. The selection range for the *δ* parameter was adjusted from 2.5–5.0 to 4.0–8.0, and the adjustment was made based on the criteria developed after testing with reference to the data.

The prediction of HFRS is widespread both domestically and internationally, with models ranging from ARIMA (19) to Holt–Winters (20) using time series analysis for the univariate prediction of HFRS, achieving good results. However, since HFRS is a natural epidemic, environmental factors greatly influence the transmission of the pathogen and the host. Therefore, this study examined the impact of meteorological factors with lag effects during different periods, classified into high and low epidemic phases using the MEM. This will help future disease control departments implement targeted preventive measures and strategies under different climatic conditions based on the epidemic intensity. We found that Liaoning province exhibited different susceptibility compared to the other regions. This finding is in agreement with the findings of several studies, which indicated that the HFRS epidemic in Liaoning province follows a bimodal pattern (21, 22). During the high epidemic period, HFRS was mainly affected by cold air, with the most susceptible population being in the 0-14-years age group. This finding is consistent with the findings of studies conducted in other regions of China (23, 24). The main reason may be that cold air increases indoor activity among young, immunocompromised individuals. Since rodents are the primary hosts of the HFRS virus, cold air also raises the likelihood of rodents entering indoor spaces, which significantly exacerbates the incidence of HFRS. Research on the impact of pollutants on diseases dates back to a survey conducted in the United States in 1964 (25). A subsequent study in the U.S. found that long-term exposure to fine particle pollution was linked to death from ischemic heart disease and stroke, highlighting the need for continued improvements in air quality to prevent cardiovascular disease (26). In the field of infectious diseases, air pollution research has primarily focused on respiratory diseases, with little attention given to natural epidemic diseases such as HFRS. A survey in Tianjin found that air pollution control efforts were primarily focused on fulfilling local responsibilities (27), highlighting the impact of air pollution on local health and diseases. Therefore, this study first explored the lagged relationship between air pollution and HFRS, identifying particulate matter (PM) as the main environmental factor. Specifically, low levels of PM_10_ and high levels of PM_2.5_ were significant at a maximum lag of 6 months, with sensitivity concentrated in the age group of 40–59 years. The reason for this may be that middle-aged individuals are more likely to overlook pollution issues during periods of high air pollution, increasing their time and chances of being exposed to environmental hazards. This, in turn, can significantly enhance exposure to pathogens and host animals. Moreover, for a transmission pathway as unique as aerosols, particulate matter may contribute to the transmission rate, although the exact mechanism remains unknown. This study also conducted a multiple regression analysis of environmental factors to explore the predictive power of machine learning. Although time variables were not included in the prediction model, as in the study by Chao Zhang et al. (24), the application of different models with varying parameters for hierarchical exploration helped reduce errors from omitted variables and increased confidence in the predictive power. The results showed better prediction accuracy in Liaoning province, which is consistent with previous findings regarding the lagged sensitivity of environmental factors. The SVM model proved to be more stable than the GPR. This also confirmed the advantage of combining the traditional ARIMA time series model with the SVM algorithm to enhance the time series model for HFRS disease prediction, as demonstrated by Chao Zhang et al. (24). However, this study focused more specifically on the northeastern region of China and did not explore the southern regions, which limited the ability to extrapolate the effects of HFRS and natural environmental factors across the country.

## 5 Conclusion

This is the first mathematically based study on the seasonal threshold of HFRS in northeastern China, enabling accurate estimation of the epidemic levels. Our findings support that long-term exposure to air pollution is a risk factor for HFRS. Therefore, we should focus on monitoring pollutants in cold conditions and developing HFRS prediction models.

## Data Availability

Publicly available datasets were analyzed in this study. This data can be found here: the National Public Health Data Centre of China (https://www.phsciencedata.cn/) and the National Oceanic and Atmospheric Administration (NOAA) (https://www.noaa.gov/).

## Footnotes

^1^ https://www.phsciencedata.cn/
