# Supplementary material for: Effect and prediction of long-term weather and pollutant exposure on hemorrhagic fever with renal syndrome: based on statistical models
Source: Front Public Health. 2025 Jan 31;13:1393763. doi: 10.3389/fpubh.2025.1393763 (PMC11825741; doi:10.3389/fpubh.2025.1393763)
Supplement: Supplementary file 1 [file Data_Sheet_1.doc]

**Supplementary material**

**Contents**

**1.Figure S1** The geographical location in Northeast China…………………………S1

**2.Figure S2** Relative risk of meteorological variables on HFRS incidence over 6 lag months, including air temperature, dew point temperature, wind direction and windspeed……………………………………………………………………………S2

**3.Figure S3** Effect of different meteorological variables on the incidence of HFRS at different months for total, regions and age groups………………………………..…S3

**4.Figure S4** Surveillance and early warning of HFRS in Northeast China during 1-12 months, 2018…………………………………………………………………………S4

**5.Figure S5** Comparison of interaction exposure-response curves of Pollutants-HFRS prevalence……………………………………………………………………………S5

**6.Table S1** Screening results of parameter δ value…………………………………. S6

**7.Table S2** Descriptive statistics of monthly HFRS cases and meteorological and pollutants factors in Northeast China………………………………………………...S7

**8.Table S3** Spearman correlation between monthly HFRS cases and meteorological factors………………………………………………………………………………...S8

**9.Table S4** Spearman correlation between monthly HFRS cases and pollutants…....S9

**10.Table S5** The cumulative effects of extreme meteorological factors on HFRS cases by region and age during the low prevalence threshold period………………….....S10

**11.Table S6** The cumulative effects of extreme meteorological factors on HFRS cases by region and age during the high prevalence threshold period……………………S11

**12.Table S7** Test of interaction model of multiple pollution factors……………….S12

**13.Table S8** Comparison of the prediction results with different kernal of gaussian distribution regression (GPR) models………………………………………………S13

**Figure S1** The geographical location in Northeast China. The map was created by ArcGIS 10.3 (Environmental Systems Research Institute; Redlands, CA, USA). The base map was acquired from the data center for geographic sciences and natural sources research, CAS (http://www.resdc.cn/data.aspx?DATAID=201).





**Figure S2** Relative risk of meteorological variables on HFRS incidence over 6 lag months, including air temperature, dew point temperature, wind direction and windspeed.

**

**

**Figure S3** Effect of different meteorological variables on the incidence of HFRS at different months for total, regions and age groups.





**Figure S4** Relative risk of pollutants on HFRS incidence over 6 lag months, including AQI, PM_2.5_ and PM_10_.





**Figure S5** Comparison of interaction exposure-response curves of Pollutants-HFRS prevalence.


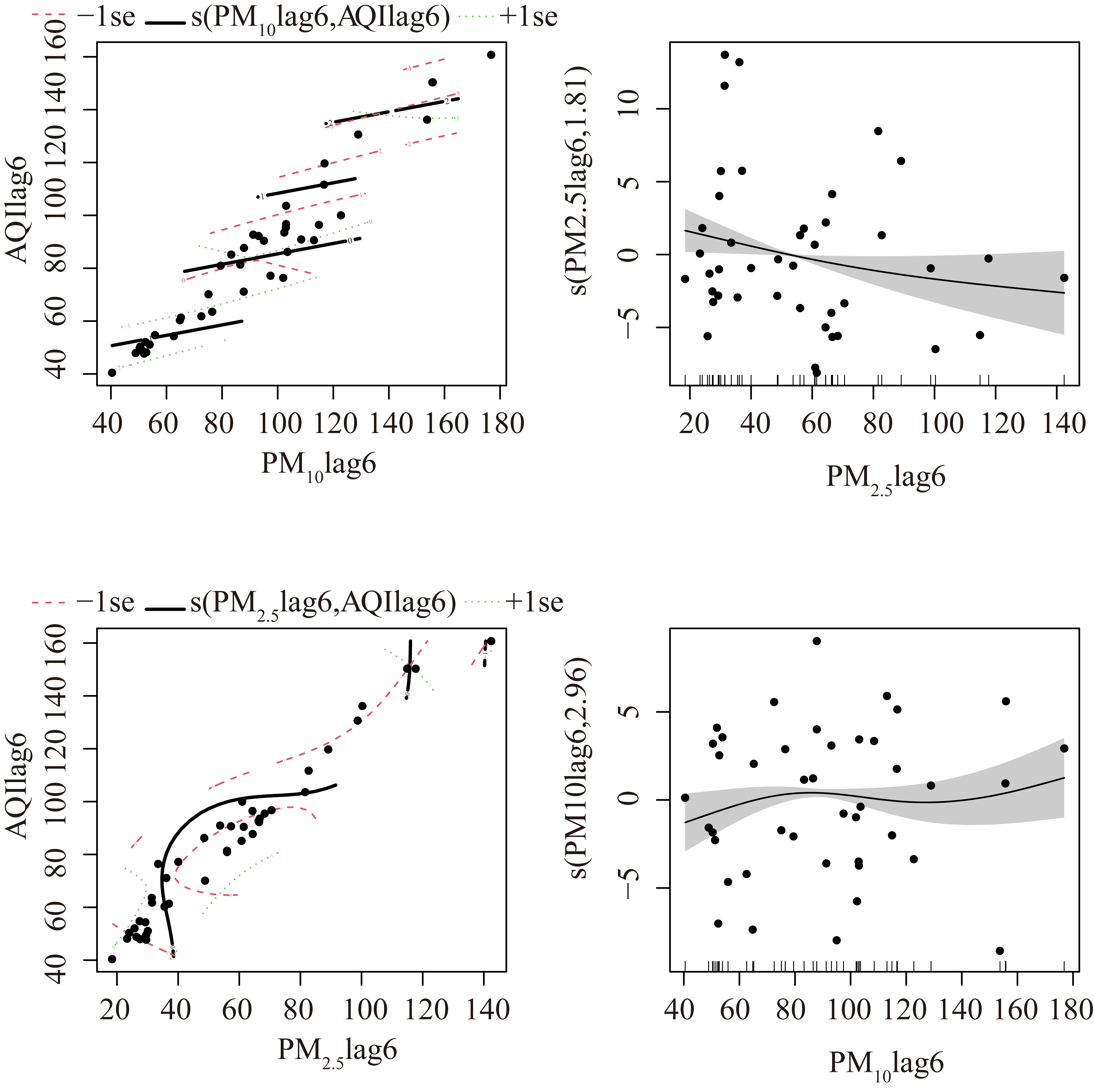


**Table S1** Screening results of parameter δ value.

| δ value | Sensitivity | Specificity | PPV | NPV | MCC | YI |
| --- | --- | --- | --- | --- | --- | --- |
| 6.5 | 0.23 | 0.94 | 0.87 | 0.38 | 0.20 | 0.16 |
| 6.6 | 0.23 | 0.94 | 0.87 | 0.38 | 0.20 | 0.16 |
| 6.7 | 0.23 | 0.94 | 0.87 | 0.38 | 0.20 | 0.16 |
| 6.8 | 0.23 | 0.94 | 0.87 | 0.38 | 0.20 | 0.16 |
| 6.9 | 0.23 | 0.94 | 0.87 | 0.38 | 0.20 | 0.16 |
| 7.0 | 0.24 | 0.94 | 0.88 | 0.38 | 0.21 | 0.17 |
| 7.1 | 0.24 | 0.94 | 0.88 | 0.38 | 0.21 | 0.17 |
| 7.2 | 0.24 | 0.92 | 0.86 | 0.38 | 0.19 | 0.16 |
| 7.3 | 0.21 | 0.95 | 0.89 | 0.37 | 0.20 | 0.15 |
| 7.4 | 0.21 | 0.94 | 0.87 | 0.37 | 0.18 | 0.14 |
| 7.5 | 0.21 | 0.94 | 0.87 | 0.37 | 0.18 | 0.14 |

PPV: Positive predictive value, NPV: Negative predictive value
MCC: Matthews correlation coefficient, YI: Youdens index

**Table S2** Descriptive statistics of monthly HFRS cases and meteorological and pollutants factors in Northeast China.

| Variables | Mean | S.D. | Min | 25th | Median | 75th | Max |
| --- | --- | --- | --- | --- | --- | --- | --- |
| Monthly HFRS cases |  |  |  |  |  |  |  |
| Total cases | 353.8 | 248.7 | 76 | 208.8 | 277.5 | 390.5 | 1690 |
| Heilongjiang | 167.1 | 140 | 24 | 82.5 | 125.5 | 199 | 1052 |
| Jilin | 78.77 | 57.92 | 14 | 46.75 | 62 | 88.25 | 357 |
| Liaoning | 107.9 | 93.38 | 20 | 58.75 | 91 | 117.5 | 643 |
| Aged 0-14 years | 5.012 | 6.08 | 0 | 2 | 3 | 6 | 32 |
| Aged 15-39 years | 138.9 | 124.7 | 14 | 65.5 | 97.5 | 151.2 | 796 |
| Aged 40-59 years | 167 | 106.2 | 41 | 101 | 136 | 190 | 710 |
| Aged >60 years | 42.93 | 23.72 | 8 | 25 | 37 | 53.25 | 152 |
| Climate parameters |  |  |  |  |  |  |  |
| Air Temperature（°C） | 5.191 | 14.21 | -24.65 | -8.728 | 7.688 | 17.44 | 25.85 |
| Dew Point Temperature（°C） | -3.048 | 14.69 | -48.57 | -15.32 | -5.365 | 10.21 | 20.87 |
| Sea Level Pressure（hpa） | 1011 | 16.66 | 908.9 | 1006 | 1013.8 | 1022 | 1031 |
| Wind Direction（Angular Degrees） | 18.45 | 2.5 | -4.175 | 17.23 | 18.718 | 19.69 | 23.07 |
| Wind Speed Rate(m/s) | 2.471 | 1.71 | -11.68 | 2.311 | 2.601 | 3.01 | 3.933 |
| Air pollution parameters |  |  |  |  |  |  |  |
| AQI | 79.58 | 31.01 | 37.92 | 51.97 | 76.79 | 93.99 | 160.7 |
| CO(μg/m^3^) | 0.963 | 0.25 | 0.614 | 0.773 | 0.8763 | 1.121 | 1.646 |
| NO_2_(μg/m^3^) | 41.81 | 9.64 | 27.55 | 34.98 | 39.59 | 47.93 | 66.38 |
| O_3_(μg/m^3^) | 55.55 | 21.54 | 23.6 | 36.24 | 54.03 | 74.28 | 96.1 |
| PM_10_(μg/m^3^) | 86.15 | 33.66 | 37.92 | 55.41 | 84.89 | 103.2 | 176.8 |
| PM_2.5_(μg/m^3^) | 52.25 | 29 | 15.43 | 29.48 | 48.7 | 66.33 | 142.4 |
| SO_2_(μg/m^3^) | 32.93 | 30.99 | 8.06 | 11.51 | 20.73 | 49.45 | 149.9 |

**Table S3** Spearman correlation between monthly HFRS cases and meteorological factors.

|  | Numbercases | Air Temperature | Dew Point Temperature | Wind Direction | Wind Speed Rate |
| --- | --- | --- | --- | --- | --- |
| Numbercases | 1.00 |  |  |  |  |
| Air Temperature | -0.18* | 1.00 |  |  |  |
| Dew Point Temperature | -0.23** | 0.88** | 1.00 |  |  |
| Wind Direction | 0.22** | -0.47** | -0.51** | 1.00 |  |
| Wind Speed Rate | 0.29** | -0.15* | -0.21* | 0.66** | 1.00 |

** P < 0.01，* P < 0.05

**Table S4** Spearman correlation between monthly HFRS cases and pollutants.

|  | Numbercases | AQI | PM_2.5_ | PM_10_ |
| --- | --- | --- | --- | --- |
| Numbercases | 1.00 |  |  |  |
| AQI | 0.40* | 1.00 |  |  |
| PM_2.5_ | 0.37* | 0.84** | 1.00 |  |
| PM_10_ | 0.40** | 0.73** | 0.92** | 1.00 |

** P < 0.01，* P < 0.05

**Table S5** The cumulative effects of extreme meteorological factors on HFRS cases by region and age during the low prevalence threshold period.

| Series | Variables | Cumulative effects(95%CI) | | | | | | | |
| --- | --- | --- | --- | --- | --- | --- | --- | --- | --- |
|  |  | Cold air effect | Hot air effect | Cold dew point effect | Hot dew point effect | Low-wind direction effect | High-wind direction effect | Windless effect | Windy effect |
|  | Total cases | 0.818(0.003,193.052) 0.848(0.033,21.497) | 1.165(0.001,2607.058) 1.303(0.000,1872532.359) | 0.425(0.002,72.138) 0.595(0.050,7.026) | 2.226(0.073,67.717) 3.846(0.001,14798.694) | 111.265(0.611,20272.679) **6.957(1.106,43.779)** | **0.438(0.202,0.951)** 0.112(0.009,1.432) | 0.267(0.005,13.473) 0.451(0.042,4.798) | 2.937(0.120,71.903) 15.135(0.005,47885.241) |
| Region | Heilongjiang | 52.252(0.070,3.9e+04) 4.134(0.077,2.2e+02) | 7.553(0.001,9.3e+04) 51.445(0.000,1.7e+09) | 0.890(0.000,1752.015) 0.911(0.024,34.882) | 1.419(0.009,235.566) 1.966(0.000,441619.238) | 430.871(0.729,254689.645) **9.316(1.007,86.171)** | 0.473(0.190,1.179) 0.206(0.010,4.179) | **0.005(0.000,0.649) 0.041(0.002,0.770)** | **74.957(1.422,3.9e+03) 53055.843(2.429,1.2e+09)** |
|  | Jilin | 0.196(0.000,1.6e+03) 0.147(0.001,2.6e+01) | 17.450(0.000,4.7e+06) 163.683(0.000,1.6e+12) | 0.001(0.000,32.402) 0.025(0.000,4.111) | 0.727(0.001,744.112) 0.046(0.000,991611.405) | 136.958(0.022,854107.621) 12.659(0.540,296.555) | **0.231(0.059,0.905) 0.009(0.000,0.798)** | 2.926(0.005,1814.812) 1.908(0.039,92.238) | 0.419(0.002,79.663) 0.113(0.000,62373.479) |
|  | Liaoning | **4.0e-05(0.000,0.165)** 0.105(0.001,12.247) | 0.000(0.000,13.826) 0.000(0.000,76.892) | 7.021(0.000,1.3e+05) 2.405(0.021,2.7e+02) | 4.306(0.006,2.9e+03) 38.464(0.000,2.5e+08) | 8.866(0.004,19914.486) 3.266(0.212,50.420) | 0.488(0.153,1.552) 0.095(0.002,4.269) | 318.784(0.989,102711.335) 32.337(0.993,1052.805) | 0.009(0.000,1.011) 0.000(0.000,1.029) |
| Age | 0-14 years | 9.0e+06(0,4.6e+26) 1.5e-02(0,1.1e+09) | 2.9e+15(0,3.4e+44) 4.0e+28(0,1.4e+82) | 1.5e-16(1.3e-41,1.9e+09) 3.0e-07(1.6e-19,5.3e+05) | **5.5e-19(10e-37,0.300) 1.8e-43(7e-85,0.047)** | 4.7e+18(0.123,1.8e+38) 7.4e+05(0.139,4.0e+12) | 0.074(0.000,30.433) 0.646(0.000,1.8e+08) | **8.3e-18(1.1e-32,0.006) 5e-11(5.4e-20,0.047)** | **8.4e+13(61.702,1.1e+26) 1.2e+35(31528.526 ,4.5e+65)** |
|  | 15-39 years | 0.620(0.000,8.7e+03) 0.442(0.002,118.920) | 4.654(0.000,3.8e+06) 15.925(0.000,1.2e+12) | 0.218(0.000,7.1e+03) 0.328(0.002,48.422) | 32.859(0.038,2.8e+04) 757.266(0.000,1.0e+10) | 135.258(0.011,1619399.953) 11.731(0.413,333.096) | 0.252(0.060,1.050) 0.013(0.000,1.414) | 0.520(0.000,686.749) 0.674(0.009,51.349) | 1.713(0.005,602.6) 3.901(0.000,1.0e+7) |
|  | 40-59 years | 0.151(0.001,16.223) 0.415(0.025,6.762) | 0.667(0.001,478.865) 0.423(0.000,74848.747) | 0.162(0.001,34.505) 0.393(0.030,5.155) | 0.897(0.025,32.646) 0.433(0.000,2534.4) | **99.001(1.142,8581.115) 5.375(1.112,25.993)** | 0.573(0.296,1.111) 0.315(0.036,2.771) | 0.225(0.008,6.245) 0.406(0.055,3.018) | 3.386(0.225,51.040) 21.661(0.023,20185.621) |
|  | 60- days | 201.056(0.007,5.9e+06) 21.737(0.054,8.8e+03) | 0.582(0.000,7.4e+05) 0.530(0.000,8.9e+10) | 142.341(0.006,3.6e+06) 16.241(0.121,2179.883) | 0.102(0.000,104.038) 0.050(0.000,7.6e+05) | 0.993(0.000,11718.549) 0.980(0.038,25.594) | 1.021(0.268,3.897) 1.086(0.013,90.479) | 0.663(0.001,710.510) 0.782(0.012,52.531) | 1.387(0.005,410.915) 2.262(0.000,3.8e+06) |

Bold font indicates statistical significance at the 0.05 level.

**Table S6** The cumulative effects of extreme meteorological factors on HFRS cases by region and age during the high prevalence threshold period.

| Series | Variables | Cumulative effects(95%CI) | | | | | | | |
| --- | --- | --- | --- | --- | --- | --- | --- | --- | --- |
|  |  | Cold air effect | Hot air effect | Cold dew point effect | Hot dew point effect | Low-wind direction effect | High-wind direction effect | Windless effect | Windy effect |
|  | Total cases | **3.5e-04(1.8e-07,0.683)** 3.485(0.026,465.406) | **7.3e-08(1.4e-13,0.039) 3.4e-13(4e-23,0.003)** | 80.531(0.000,1.6e+07) 0.442(0.009,21.848) | **4.5e+05(6.531,3.0e+10) 3.2e+09(26.040,3.9e+17)** | 1.164(0.022,61.319) 1.404(0.270,7.292) | 0.657(0.240,1.797) 0.076(0.000,19.860) | 0.533(0.007,41.87) 0.672(0.048,9.486) | 1.829(0.043,77.646) 3.605(0.001,8870.010) |
| Region | Heilongjiang | 0.001(0.000,205.734) 17.618(0.005,6.2e+04) | 0.000(0.000,8.248) 0.000(0.000,33.694) | 16.295(0.000,9.2e+09) 0.133(0.000,66.216) | 3.0e+07(0.657,1.3e+15) 2.4e+12(0.302,1.9e+25) | 0.032(0.000,28.944) 0.417(0.026,6.774) | 0.829(0.163,4.217) 0.055(0.000,386.219) | 0.029(0.000,38.582) 0.109(0.001,8.508) | 27.862(0.059,1.3e+04) 1151.431(0.003,4.3e+08) |
|  | Jilin | 0.003(0.000,5.8e+04) 0.031(0.000,1.8e+03) | 1.811(0.000,1.1e+13) 2.073(0.000,3.3e+22) | 15401.804(0.065,3.7e+09) 13.458(0.223,8.1e+02) | 11.709(0.000,2.1e+06) 0.389(0.000,1.3e+11) | 0.347(0.000,1262.782) 0.511(0.016,16.788) | 1.674(0.178,15.709) 15.116(0.000,3.9e+06) | 7.855(0.000,2.1e+05) 3.637(0.007,1794.31) | 0.144(0.000,953.8) 0.017(0.000,1.5e+06) |
|  | Liaoning | 0.002(0.000,11462.669) 23.074(0.002,3.4e+05) | 0.000(0.000,2329.633) 0.000(0.000,6.6e+05) | 36.701(0.001,1.5e+06) 0.375(0.011,12.415) | **2.7e+05(12.899,5.8e+09) 1.3e+09(81.674,2.0e+16)** | 357.904(0.169,7.6e+05) 23.375(0.929,588.083) | 0.131(0.016,1.076) 0.000(0.000,7.840) | 0.101(0.000,571.877) 0.259(0.001,48.993) | 6.061(0.004,1.0e+04) 39.803(0.000,2.1e+08) |
| Age | 0-14 years | 2.7e+04(0.000,2.4e+18) **3.2e+17(8.4e+08,1.2e+26)** | 0.000(0.000,0.000) 0.000(0.000,0.000) | 5.7e+06(2.0e-03,1.4e+16) 2.0e-03(0.000,3.651) | **2.6e+28(5.8e+17,1.1e+39) 2.4e+47(5.8e+29,9.9e+64)** | **1.2e+11(5.8e+04,2.4e+17) 6.6e+04(161.608,2.7e+07)** | **6.0e-03(0.000,0.370)** 0.000(0.000,10.674) | **9.3e-11(5.4e-19,0.016) 8.2e-07(8e-12,0.085)** | **4.1e+08(30.052,5.6e+15) 8.6e+17(1122.052,6.6e+32)** |
|  | 15-39 years | 4.130(0.000,3.5e+05) 704.905(0.434,1.1e+06) | 0.000(0.000,22.829) 0.000(0.000,294.516) | 4.4e+03(0.000,1.9e+11) 0.727(0.003,172.641) | **4.0e+07(7.629,2.1e+14) 9.0e+12(44.734,1.8e+24)** | 0.735(0.002,293.758) 1.441(0.117,17.679) | 0.522(0.114,2.397) 0.014(0.000,60.401) | 0.001(0.000,1.285) 0.018(0.000,1.100) | **342.552(1.015,1.2e+05) 2.1e+05(1.127,3.8e+10)** |
|  | 40-59 years | **1.4e-05(3.6e-10,0.598)** 2.037(2.5e-04,237.540) | 0.000(0.000,90.803) 0.000(0.000,1660.044) | 280.993(0.001,9.4e+07) 1.126(0.019,66.144) | 1.8e+04(0.147,2.1e+09) 1.9e+07(0.064,5.9e+15) | 1.815(0.007,468.901) 1.291(0.130,12.840) | 0.891(0.220,3.606) 0.652(0.000,1491.351) | 14.012(0.033,5919.761) 5.066(0.129,198.348) | 0.095(0.001,17.145) 0.007(0.000,359.967) |
|  | 60- days | **6.6e-11(3.5e-15,1.2e-06)** 0.072(1.1e-04,47.846) | **6.5e-14(1e-21,4.1e-06) 5.8e-24(1.7e-37,1.9e-10)** | 0.000(0.000,182.309) **0.003(0.000,0.566)** | 2.5e+06(0.357,1.7e+13) 5.4e+09(0.023,1.3e+21) | 1.582(0.008,311.240) 3.571(0.403,31.646) | **0.197(0.052,0.746) 4.5e-05(2.8e-08,0.072)** | 3.106(0.013,729.374) 1.955(0.071,53.489) | 0.405(0.004,43.634) 0.157(0.000,2667.953) |

Bold font indicates statistical significance at the 0.05 level.

**Table S7** Test of interaction model of multiple pollution factors.

| Indicator | Interaction | Parameters | edf | Ref.df | χ^2^ | P | R^2^(adj) | Deviance explained | GCV |
| --- | --- | --- | --- | --- | --- | --- | --- | --- | --- |
| Pollutants-Pollutants | s(PM_10_,AQI) | PM_10_, AQI | 2 | 2 | 3.475 | **0.0431*** | 0.239 | 42.30% | 41.901 |
|  |  | PM_2.5_ | 1.805 | 1.962 | 3.17 | 0.0758 |  |  |  |
|  | s(PM_2.5_,AQI) | PM_2.5_, AQI | 2.935 | 2.995 | 5.137 | **0.00643**** | 0.441 | 58.00% | 34.905 |
|  |  | PM_10_ | 2.956 | 2.997 | 5.802 | **0.00359**** |  |  |  |

** P < 0.01，* P < 0.05

Bold font indicates statistical significance at the 0.05 level.

**Table S8** Comparison of the prediction results with different kernal of gaussian distribution regression (GPR) models.

| Model | Series | | Parameters | cv.fold | Training set | | |  | Test set | | |
| --- | --- | --- | --- | --- | --- | --- | --- | --- | --- | --- | --- |
|  |  |  |  |  | RMSE | R^2^ | MAE |  | RMSE | R^2^ | MAE |
| GPR (rbfdot) |  | Total cases | sigma=0.2999 | 10 | 70.200 | 0.670 | 54.119 |  | 63.468 | 0.245 | 51.828 |
|  | Region | Heilongjiang | sigma=0.2999 | 10 | 46.771 | 0.710 | 33.387 |  | 44.439 | 0.087 | 37.366 |
|  |  | Jilin | sigma=0.2999 | 10 | 12.568 | 0.736 | 9.653 |  | 13.673 | 0.065 | 10.621 |
|  |  | Liaoning | sigma=0.2999 | 10 | 18.557 | 0.680 | 15.873 |  | 28.662 | 0.483 | 24.445 |
|  | Age | 0-14 years | sigma=0.2999 | 10 | 1.691 | 0.467 | 1.305 |  | 1.959 | 0.018 | 1.411 |
|  |  | 15-39 years | sigma=0.2999 | 10 | 19.077 | 0.700 | 15.625 |  | 14.398 | 0.540 | 12.272 |
|  |  | 40-59 years | sigma=0.2999 | 10 | 36.359 | 0.674 | 28.654 |  | 36.848 | 0.176 | 29.421 |
|  |  | 60- days | sigma=0.2999 | 10 | 16.605 | 0.604 | 11.804 |  | 16.706 | 0.097 | 12.976 |
| GPR (polydot) |  | Total cases | degree=1,scale=1,offset=1 | 10 | 85.916 | 0.323 | 64.628 |  | 77.284 | 0.082 | 64.267 |
|  | Region | Heilongjiang | degree=1,scale=1,offset=1 | 10 | 56.213 | 0.328 | 41.499 |  | 53.052 | 0.009 | 42.697 |
|  |  | Jilin | degree=1,scale=1,offset=1 | 10 | 15.604 | 0.402 | 12.017 |  | 14.378 | 0.094 | 11.310 |
|  |  | Liaoning | degree=1,scale=1,offset=1 | 10 | 21.399 | 0.478 | 16.304 |  | 29.816 | 0.339 | 23.137 |
|  | Age | 0-14 years | degree=1,scale=1,offset=1 | 10 | 2.029 | 0.134 | 1.560 |  | 1.595 | 0.388 | 1.269 |
|  |  | 15-39 years | degree=1,scale=1,offset=1 | 10 | 23.886 | 0.340 | 18.831 |  | 20.268 | 0.169 | 16.510 |
|  |  | 40-59 years | degree=1,scale=1,offset=1 | 10 | 42.893 | 0.371 | 32.690 |  | 41.886 | 0.102 | 35.376 |
|  |  | 60- days | degree=1,scale=1,offset=1 | 10 | 20.758 | 0.183 | 14.209 |  | 18.254 | 0.010 | 13.935 |
| GPR (laplacedot) |  | Total cases | sigma=0.2999 | 10 | 70.370 | 0.732 | 54.713 |  | 63.941 | 0.231 | 53.432 |
|  | Region | Heilongjiang | sigma=0.2999 | 10 | 47.554 | 0.764 | 34.631 |  | 44.156 | 0.088 | 36.767 |
|  |  | Jilin | sigma=0.2999 | 10 | 13.138 | 0.795 | 10.040 |  | 13.590 | 0.055 | 10.363 |
|  |  | Liaoning | sigma=0.2999 | 10 | 18.283 | 0.741 | 15.486 |  | 28.575 | 0.492 | 24.023 |
|  | Age | 0-14 years | sigma=0.2999 | 10 | 1.633 | 0.620 | 1.266 |  | 1.869 | 0.061 | 1.378 |
|  |  | 15-39 years | sigma=0.2999 | 10 | 19.395 | 0.755 | 15.970 |  | 15.718 | 0.454 | 13.254 |
|  |  | 40-59 years | sigma=0.2999 | 10 | 36.240 | 0.731 | 28.819 |  | 36.697 | 0.176 | 29.906 |
|  |  | 60- days | sigma=0.2999 | 10 | 16.493 | 0.707 | 11.803 |  | 16.335 | 0.110 | 12.464 |
| GPR (besseldot) |  | Total cases | sigma=1,order=1,degree=1 | 10 | 80.967 | 0.466 | 62.522 |  | 57.054 | 0.395 | 48.174 |
|  | Region | Heilongjiang | sigma=1,order=1,degree=1 | 10 | 53.811 | 0.494 | 38.250 |  | 40.670 | 0.231 | 34.531 |
|  |  | Jilin | sigma=1,order=1,degree=1 | 10 | 15.048 | 0.530 | 11.658 |  | 12.600 | 0.171 | 9.397 |
|  |  | Liaoning | sigma=1,order=1,degree=1 | 10 | 21.246 | 0.523 | 17.898 |  | 26.971 | 0.593 | 21.946 |
|  | Age | 0-14 years | sigma=1,order=1,degree=1 | 10 | 1.871 | 0.291 | 1.442 |  | 1.881 | 0.067 | 1.373 |
|  |  | 15-39 years | sigma=1,order=1,degree=1 | 10 | 22.069 | 0.514 | 17.928 |  | 13.505 | 0.643 | 11.596 |
|  |  | 40-59 years | sigma=1,order=1,degree=1 | 10 | 41.761 | 0.470 | 33.403 |  | 33.913 | 0.296 | 27.662 |
|  |  | 60- days | sigma=1,order=1,degree=1 | 10 | 19.211 | 0.356 | 13.608 |  | 14.723 | 0.281 | 11.117 |
